# Reviews of Books

**Published:** 1922-05

**Authors:** 


### REVIEWS OF BOOKS.

**A CENTURY OF MEDICINE AT PADUA.** By Sir George Newman. (British Periodicals, Ltd.; 1/- net.)

Last spring Sir George Newman paid a visit to Padua, where he studied the academic past of the first medical school in Italy. In the thirteenth century this mother of medicine "blazed the trail" for the grand sweep of the Renaissance. Byzantium had conserved Greek medicine, and Italian ships that carried there the fruits of Lombardy brought back manuscripts and drugs. Thus Padua, the University of Venice, was equipped with Greek learning, and secured by the generosity of the Venetian merchants independence of thought and study. There came Thomas Linacre, the founder of the College of Physicians in London, and took his degree as a Doctor of Medicine. Forty years later Edward Wootton followed his example, and subsequently became physician to Henry VIII. William Harvey, physician to Charles I., was at Padua probably in 1600. All these famous men travelled over the Alps by packhorse, and suffered abundant discomforts in towns and monasteries on the journey in order to drink of knowledge at Italian sources. At Padua the whole human body was twice dissected in public each year by the professor of anatomy, and on these occasions the attendance was so large that a sort of wooden barrack was built to serve as a theatre. Among the prophets of medicine there was Vesalius, a young Belgian with an English mother. He studied nature and not books, and so a new epoch began, the beginning not only of modern anatomy, but of modern medicine. Fracastorius, another forerunner, first opened his eyes to the nature of contagion. Fabricius enabled Harvey to discover the truth of the circulation of the blood. All these historical events Sir George Newman describes with skill and charm, and Lady Newman adds to the delight of her husband's work by several delicate sketches. This booklet is a "Song of man's inheritance, of his unconquerable mind, of his partnership with the eternal wisdom."—B. S. T.

**A MANUAL OF SELECTED BIOCHEMICAL METHODS.** By Frank P. Underhill, Ph.D. (Chapman & Hall; 17s. 6d. net.)

Just as classical scholars have found it difficult to arrive at an unanimous opinion upon the importance of retaining the humanities in present-day education at schools and at the Universities, so have medical men found it difficult to determine the choice of obligatory subjects in the student's curriculum. In many teaching centres it is apparent that purely scientific methods are largely replacing the exercise of critical clinical observation, and final-year men are spending in laboratories time which for most of them could be spent more profitably in the ward. Medical schools recruit for a highly arduous profession, of which the ramifications are yearly becoming deeper and more interwoven; a profession which the majority of men embrace as a means of livelihood. Thus their equipment should be such that they can at once begin on their own account, after quitting Hospital work for which they have all along been specially trained, and this equipment should consiste of a minimum of laboratory apparatus, with a maximum grasp of the principles underlying the elaborate methods that modern investigations have shown to be most useful in recognising the presence of disease, dealing with its declared manifestations and assessing the rate of its progress.

The general practitioner is recognised now more than ever as the pivot upon which must hang the main workings of a perfectly efficient public health service; his ubiquity, his close association with persons in their native surroundings, and his intimate contact with the beginnings of disease, render it important that he shall start endowed with a broad perspective of his future work, imbued with a sense of its importance. He must rough-hew his living material to make the outlines clearly discernible as those of the ideal to which preventive medicine aspires. He must labour with sweat on his brow, as a Rodin would, with blood and clay and sand, leaving the expression of Canova's pure grace to the crystalline genius of more favoured brethren.

It is essential for the continued vitality of our profession that some men should devote themselves, in the hallowed groves of the University, to an academic career which can keep abreast of the advance in scientific knowledge; one in which there is the time and opportunity to comprehend the significance of facts elucidated in laboratories commanding every available refinement of method, and to correlate the findings with their own observance or deranged and faulty physiological processes or emphatically pathological states.

The resources of the biochemist and the bacteriologist are being brought nearer and nearer to the poor through—to name one agency only—a wide extension of public health clinic systems. The craving of a sick man for relief, a speedy cure and a name for his complaint can still be satisfied, in part or whole, by the practitioner, whilst the demand for material by the laboratory is being met, and our conceptions of the cause, extent and result of a disease quickened. But the findings of the laboratory workers will be of use neither to the doctor nor the patient unless they can be assimilated to the circumstances of the particular case. To strain an analogy, they are anti-bodies—the antigen is in the care of the clinician, who should realise that value can be ascribed to them only when *he* uses his "substance sensibilitatrice" and arrives at an accurate diagnosis, or prognosis, or directs the line of treatment.

The admirable book before us does not embark upon considerations which should properly be left to the medical man. Dr. Underhill has compiled an excellent laboratory manual of biochemical methods in the examination of urine, blood and gastric contents, not claiming any originality for the work. The methods are those which have stood the test of time; the wording is concise, the letterpress clear, the explanation of the few diagrams easy to follow. We can find no error in the directions for carrying out the tests, which are well arranged and prefaced by a declaration of the principle of each.

In no spirit of carping criticism, we would point out that no mention is made of Carnot's method of collecting gastric juice for analysis; probably Dr. Underhill has not arrived at a definite conclusion as to its value over the Rehfuss tube, and he is to be congratulated upon having disregarded methods or improvements of methods which have not been duly weighed as to their merits by the mass of authoritative opinion in biochemistry.

There is an excellent index, a refreshing freedom from annoying cross-references at the foot of the pages, and a sound foreword, which every laboratory worker should read. Altogether, this is a manual we can thoroughly recommend as one valuable to the student, and invaluable to the house-physician for carrying out those duties physicians will expect from him.—E. L. W.

---

**INVESTIGATION ON SOIL POLLUTION AND THE RELATION OF THE VARIOUS TYPES OF PRIVIES TO THE SPREAD OF INTESTINAL INFECTIONS.** By I. J. Kligler, Ph.D. (The Rockefeller Institute of Medical Research.)

The relation of soil pollination to the spread of infectious disease has been a fallow field to sanatarians since the time of Pettenkofer. It is true that several investigators have lightly cultivated small areas, but the greater part is still untilled.

One of the most recent inquiries into the matter is that conducted by Dr. Kligler, who has approached the subject from the experimental and practical standpoints. In the laboratory repeated tests were made to determine:—

(1) The viability of typhoid and dysentery baccilli in soil and in excrement under different conditions.

(2) Their ability to penetrate columns of soil of different porosity.

(3) Their viability in septic fluids and effluents.

(4) The nature of the antagonistic factors in soil and septic material which influence the viability of these micro-organisms.

In field works various types of privies were examined with regard to—

(1) The extent of the pollution of the soil surrounding these privies.

(2) Their relation to well pollution.

(3) The drainage of the material from the privies through the soil.

The following facts were brought to light:—

(1) The typhoid and dysentery bacilli succumbed rapidly on exposure to an unnatural environment; in septic tanks the organisms died in 1 to 5 days.

(2) The spread of pollution from a focal point was limited in scope; in dense soil bacilli failed to penetrate through 1 foot of soil.

(3) The pollution of wells is usually surface in origin.

The practical value of these results seems to be that pits and privies *if properly constructed* are not likely to cause serious contamination *of the soil*, but the medical officer of health will still be faced with problem of how to prevent faecal contamination of food by flies, dust and other agencies.

The experiments outlined in this monograph, valuable as they are, should not stay for one moment the conversion of privies and pail closets to the water carriage system. The slime pits of Lancashire have already cost that county dear in infant lives.—J. C.

